# Inhibition of FGFR2-Signaling Attenuates a Homology-Mediated DNA Repair in GIST and Sensitizes Them to DNA-Topoisomerase II Inhibitors

**DOI:** 10.3390/ijms21010352

**Published:** 2020-01-05

**Authors:** Boichuk Sergei, Dunaev Pavel, Galembikova Aigul, Bikinieva Firyuza, Nurgatina Ilmira, Mustafin Ilshat, Aukhadieva Aida, Kurtasanov Refat, Andriutsa Natalia, Shagimardanova Elena, Gorbunova Vera

**Affiliations:** 1 Department of Pathology, Kazan State Medical University, Kazan 420012, Russia; dunaevpavel@mail.ru (D.P.); ailuk000@mail.ru (G.A.); firuza1995@mail.ru (B.F.); ilmiranurgatina@gmail.com (N.I.); arom1705@mail.ru (A.A.); 2 Department of Biochemistry, Kazan State Medical University, Kazan 420012, Russia; ilshat64@mail.ru; 3 Tatarstan Cancer Center, Kazan 420029, Russia; kurt-asan@mail.ru; 4 Department of Pathophysiology, I.M. Sechenov First Moscow State Medical University, Moscow 119146, Russia; natiandriutsa@mail.ru; 5 Institute of Fundamental Medicine and Biology, Kazan (Volga Region) Federal University, Kazan 420008, Russia; rjuka@mail.ru; 6 Department of Biology, University of Rochester, Rochester, NY 14627, USA; vgorbuno@ur.rochester.edu

**Keywords:** gastrointestinal stromal tumor cells (GIST), imatinib mesylate (IM), resistance, FGFR-signaling, Rad51 recombinase, homology-mediated DNA repair, DNA-topoisomerase II inhibitors

## Abstract

Deregulation of receptor tyrosine kinase (RTK)-signaling is frequently observed in many human malignancies, making activated RTKs the promising therapeutic targets. In particular, activated RTK-signaling has a strong impact on tumor resistance to various DNA damaging agents, e.g., ionizing radiation and chemotherapeutic drugs. We showed recently that fibroblast growth factor receptor (FGFR)-signaling might be hyperactivated in imatinib (IM)-resistant gastrointestinal stromal tumors (GIST) and inhibition of this pathway sensitized tumor cells to the low doses of chemotherapeutic agents, such as topoisomerase II inhibitors. Here, we report that inhibition of FGFR-signaling in GISTs attenuates the repair of DNA double-strand breaks (DSBs), which was evidenced by the delay in γ-H2AX decline after doxorubicin (Dox)-induced DNA damage. A single-cell gel electrophoresis (Comet assay) data showed an increase of tail moment in Dox-treated GIST cells cultured in presence of BGJ398, a selective FGFR1-4 inhibitor, thereby revealing the attenuated DNA repair. By utilizing GFP-based reporter constructs to assess the efficiency of DSBs repair via homologous recombination (HR) and non-homologous end-joining (NHEJ), we found for the first time that FGFR inhibition in GISTs attenuated the homology-mediated DNA repair. Of note, FGFR inhibition/depletion did not reduce the number of BrdU and phospho-RPA foci in Dox-treated cells, suggesting that inhibition of FGFR-signaling has no impact on the processing of DSBs. In contrast, the number of Dox-induced Rad51 foci were decreased when FGFR2-mediated signaling was interrupted/inhibited by siRNA FGFR2 or BGJ398. Moreover, Rad51 and -H2AX foci were mislocalized in FGFR-inhibited GIST and the amount of Rad51 was substantially decreased in -H2AX-immunoprecipitated complexes, thereby illustrating the defect of Rad51 recombinase loading to the Dox-induced DSBs. Finally, as a result of the impaired homology-mediated DNA repair, the increased numbers of hypodiploid (i.e., apoptotic) cells were observed in FGFR2-inhibited GISTs after Dox treatment. Collectively, our data illustrates for the first time that inhibition of FGF-signaling in IM-resistant GIST interferes with the efficiency of DDR signaling and attenuates the homology-mediated DNA repair, thus providing the molecular mechanism of GIST’s sensitization to DNA damaging agents, e.g., DNA-topoisomerase II inhibitors.

## 1. Introduction

Receptor tyrosine kinase (RTK) signaling is known to be activated upon DNA damage and in turn, activates DNA damage response (DDR) signaling networks to restore genome integrity and maintain cellular homeostasis. Some of the RTKs become activated not only in the response to the extracellular signals originating from the binding of the corresponding ligands and growth factors, but also during DNA damage independently of the ligand binding [1,2,3,4]. RTK activation during DNA damage is associated with their phosphorylation and allows the interactions between RTK with the multiple DNA damage signaling and repair (DDR) proteins, thereby illustrating the intensive cross-talk between RTKs and DDR signaling pathways over DNA damaging conditions. For example, epidermal growth factor receptor (EGFR) became phosphorylated at residues Y845 and Y1173 after exposure to ionizing radiation, became internalized into the nuclear compartment (as a component of lipid rafts) to interact and modulate activity of DNA-PK, a key component of non-homologous end-joining (NHEJ) pathway involved in DNA double-strand breaks (DSBs) repair [5]. Inhibition of EGFR’s nuclear import by cetuximab (Erbitux) increases the radiosensitivity of cancer cells by suppressing DNA-PK activity, thereby illustrating the direct regulation of NHEJ-mediated repair by EGFR [6]. Similarly, gefitinib (Iressa), an EGFR small molecule inhibitor, attenuated DNA repair in lung cancer cells exposed to ionizing radiation, thereby revealing an ability of EGFR signaling to modulate DDR in cancer cells during an external DNA damage [7]. Besides the EGFR-mediated pathway, insulin-like growth factor 1 receptor (IGF-1R) - signaling was also shown to be actively involved in the modulation of multiple DDR pathways, particularly involved in DNA DSBs repair. Again, activation of IGF-1R signaling was also observed in cancer cells exposed to ionizing radiation and depletion of IGF-1R or pharmacological inhibition of IGF-signaling increased cellular radiosensitivity by attenuating DSBs repair by homologous recombination (HR) and NHEJ, as well [4,8,9]. Given that IGF-1R activation leads to the disruption of the complex composed of insulin receptor substrate 1 (IRS-1) and recombinase Rad51, thereby promoting Rad51 entry into the nucleus, inhibition of IGF-1R prevents Rad51 nuclear translocation and compromises HR-mediated DNA repair [10]. Additionally, inhibition/depletion of IGF-1R attenuates the radiation-induced Ku86 binding to DNA, thereby enhancing the radiosensitivity of lung cancer cells due to the interruption of NHEJ-mediated DSBs repair mechanisms [4].

Fibroblast growth factors (FGF) and their receptors (FGFR1-4) are known as the potent regulators of a broad spectrum of physiologic processes, such as cellular differentiation, proliferation, survival, migration, and angiogenesis [11,12]. At the other hand, FGFR signaling pathways frequently become dysregulated (in most of the cases, overactivated) in the multiple human malignancies, thereby making this pathway an attractive molecular target for cancer therapy. Dysregulation of FGFR-mediated pathways in human malignancies typically include FGFR activating somatic mutations and/or amplifications thereby leading to ligand-independent FGFR signaling to promote cancer cell proliferation and survival [13,14]. In addition to FGFR molecular alterations, multiple aberrant autocrine and paracrine FGFRs/FGFs loops have been also described in several cancer models, including endometrial, bladder, non-small cell lung cancer, breast, multiple myeloma, prostate, gastric and colon cancers [13,15]. The activation of FGFR signaling in cancer cells might also provide direct and/or indirect (e.g. vascular endothelial growth factor receptor (VEGFR) and platelet-derived growth factor receptor (PDGFR)-mediated) pro-angiogenic effects, thus facilitating tumor development, disease progression, and drug resistance. Indeed, we found that an acquired resistance of gastrointestinal stromal tumors (GIST) to imatinib mesylate (IM) might be due to activation of the FGFR signaling pathway [16]. Strikingly, inhibition of FGF-signaling in IM-resistant GISTs restored their sensitivity to IM in vivo and in vitro [17]. We also observed that inhibition of FGFR signaling in IM-resistant GIST cells sensitizes them to DNA damaging agents, such as topoisomerase II inhibitors. Importantly, when FGFR2 was knocked down by siRNA and/or inhibited with BGJ398, a selective FGFR1-4 inhibitor, IM-resistant GIST cells exhibited a reduced level of Rad51 recombinase after doxorubicin exposure, thus suggesting the attenuation of homology-mediated DNA repair mechanisms [18].

To corroborate these findings, we examined the molecular mechanisms responsible for GIST sensitization to DNA damaging agents after FGFR depletion and inhibition. We found that the knockdown/inhibition of FGFR-signaling in GISTs substantially attenuated DNA DSBs repair, which was evidenced by a substantial delay in γ-H2AX decline after doxorubicin (Dox)-induced DNA damage and sustained DNA damage assessed by a Comet assay. By utilizing the GFP-based reporter constructs, we observed that FGFR inhibition substantially attenuated HR-mediated DSBs repair in GISTs and have no impact on NHEJ-pathway after DSBs induction. Of note, BGJ398, a selective FGFR inhibitor, did not reduce the number phospho-RPA foci following doxorubicin treatment, suggesting no impact on the processing of DSBs. Similarly, FGFR inhibition/depletion in GISTs did not affect the numbers of BrdU foci that were highly co-localized with pRPA foci in GIST cells treated with Dox, thereby suggesting the generation of ssDNA foci and RPA loading to ssDNA are ongoing normally in these experimental conditions. However, the numbers of Rad51 foci were significantly decreased after FGFR inhibition. Moreover, Rad51 foci were mis-localized with H2AX foci, therefore suggesting the defect of Rad51 recombinase loading to the DSBs sites and explaining the potential mechanism responsible for attenuation of HR-mediated DSBs repair.

Therefore, overactivation of the FGF-signaling pathway in IM-resistant GIST might be considered as a prospective molecular target to enhance their sensitivity to certain chemotherapeutic agents (e.g., DNA-topoisomerase II inhibitors) inducing DNA DSBs.

## 2. Results

### 2.1. FGFR Inhibition Delays DNA DSBs Repair IM-Resistant GISTs

It was shown previously that GIST cell lines and patient-derived IM-resistant primary GIST cells are sensitive to certain chemotherapeutic agents, such as topoisomerase II inhibitors and transcriptional inhibitors [19,20]. Based on our previous data illustrating that inhibition of FGF-signaling in GIST effectively sensitized them to DNA damaging agents (e.g., topoisomerase II inhibitors) [18], we sought to examine whether it might be due to decreased efficiency of DDR mechanisms involved in repair of the double-strand breaks (DSBs).

To assess whether inhibition of FGFR-signaling has an impact on DDR in GISTs, IM-naïve (T-1) vs. resistant (T-1R) cells were treated with Dox (0.5 μg/mL) for 2 h, after which the drug-containing media was removed and cells were further cultured without drug in absence (control) or presence of BGJ398, a selective FGFR1-4 inhibitor, for 48 h to assess an efficiency of DNA repair of Dox-induced DNA damage. Formation of DNA DSBs in Dox-treated GIST was assessed by a single cell electrophoresis (a Comet assay) and expression of histone H2AX (γ-H2AX) phosphorylated at Ser139 residues, a well-known marker for DSBs, was also used for these experimental settings.

**Figure 1:**
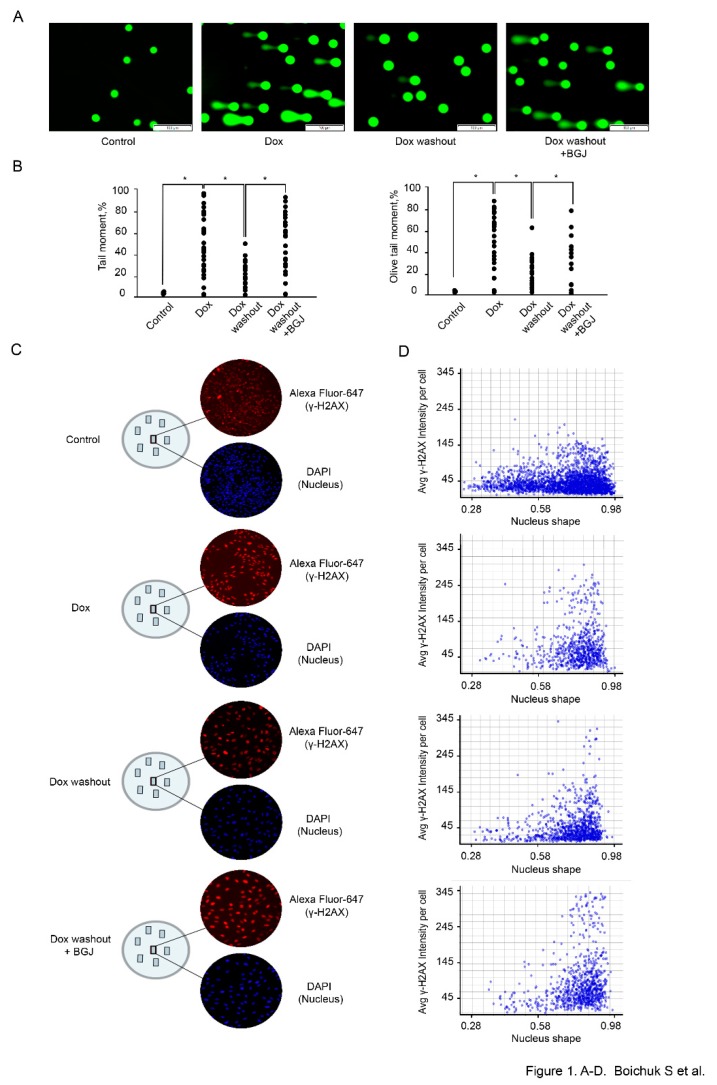
Inhibition of FGF-signaling delays the kinetics of γ-H2AX decline in doxorubicin-treated GIST cells. GIST T-1 cells treated with solvent (control) and doxorubicin (dox) 0.5 μg/mL for 2 h following washout for 8 h and culture in the absence (Dox washout) and presence of BGJ398 (1 µM), an FGFR1-4 inhibitor (Dox washout + BGJ398). (**A**) Representative images of comets from theWe added experimental settings indicated above (Scale bars = 100 μm). (**B**) Graphic depiction of the calculated Tail Moment and Olive Tail Moment from analysis of alkaline Comet Assay shown in Figure 1A. Data is shown for a representative experiment, where at least 50 comets were quantitated for each experimental condition. (**C**) Cells were grown on slides for 24 h and treated with Dox and BGJ398 as indicated above. Cells were fixed and stained with DAPI (blue) and γH2AX-specific antibody (red). γH2AX-specific fluorescence intensity was measured for each nucleus (DAPI) and quantified automatically. (**D**) Histograms of γ-H2AX-specific fluorescence at the single-nucleus level. All images were acquired by GE Cytell imager as described in “Materials and methods”.

We observed a significant increase of the tail moment (TM) and Olive tail moment (OTM) in GIST T-1 cells treated with Dox (0.5 μg/mL) for 2 h (Figure 1A,B). A substantial decrease of both parameters indicated above was found in GIST T-1 cells on day 2 after Dox washout, thus revealing an effective repair of DNA DSBs at this time period (Figure 1A,B). Strikingly, a significant increase of the TM and OTM was observed in Dox-treated GIST T-1 cells cultured after Dox washout in presence of BGJ398 when compared to control cells (Figure 1A,B), thereby illustrating that inhibition of FGF-signaling attenuates the repair of Dox-induced DSBs. Similar effects were observed for GIST T-1R cells (data is not shown).

Given that formation and resolution of γ-H2AX foci are commonly used to assess the efficiency of DNA DSBs repair, we examined the expression of this marker by TexRed-labeled immunofluorescence staining. DAPI-stained nuclei and γ-H2AX-specific fluorescence were automatically quantified at the single nucleus level by using a specific algorithm shown in “Materials and methods”. The analysis of the signal intensity at the single nucleus level revealed a substantial increase of γ-H2AX-specific fluorescence in IM-resistant GIST after Dox treatment when compared to control non-treated cells (Figure 1C). As expected, the signal intensity of γ-H2AX-specific fluorescence was substantially decreased at 48 h after Dox was washed out, thereby revealing an effective repair of Dox-induced DNA DSBs (Figure 1C). Again, inhibition of FGF-signaling in GIST leads to the retention of γ-H2AX-specific fluorescence after Dox washout when comparted to Dox-treated cells cultured alone after the drug removal (Figure 1C). Histograms of γ-H2AX-specific fluorescence at the single-nucleus level also illustrated a similar pattern (Figure 1D), thereby revealing a significant impact of FGFR-signaling on DDR in GISTs. Similar results were obtained for IM-naïve GIST (data is not shown).

All together, these results implicate that inhibition of FGF-signaling interferes with the efficiency of DNA repair and cell-cycle regulation in GIST, thereby suggesting the potential mechanism of GIST’s sensitization to topoisomerase II inhibitors.

### 2.2. FGFR Inhibition Impairs DNA Damage Repair (DDR) and Signaling in GISTs

Given that DSBs repair involves two distinct DDR pathways, we examined the impact of FGFR inhibition on the HR and NHEJ repair pathways by utilizing GFP-based reporter constructs, having *GFP* gene containing recognition sites for a I-SceI endonuclease for induction of DSBs. Since GFP gene is inactivated by an additional exon (NHEJ reporter cassette), or by mutations (HR reporter cassette), these constructs are initially GFP-negative, whereas, the successful repair of the I-SceI-induced breaks by NHEJ or HR restores the functional GFP gene. Thus, the quantification of the number of GFP- positive cells by flow cytometry provides a quantitative measure of NHEJ or HR efficiency [16].

To examine whether inhibition of FGF-signaling attenuates DSBs repair in GIST cells, HR- and NHEJ-expressing GIST cells were previously generated according to the published protocol [21]. The cells exhibiting the reporter constructs were transfected with pCBASceI or empty vector plasmids to introduce DSBs. The cells were simultaneously transfected with 0.1 μg pDsRed2-N1 as a transfection efficiency control. Four days post-transfection, the cells were analyzed by flow cytometry to count the numbers of GFP- and DsRed-positive cells. The efficiency of HR and NHEJ was calculated as a ratio of GFP+/DsRed+ cells.

**Figure 2:**
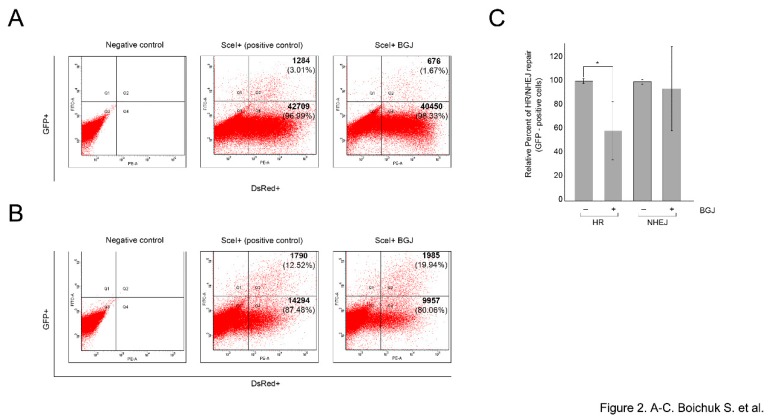
FGFR inhibition attenuates homology-mediated (HR) DNA repair in GIST. IM-resistant GIST-T1-HR (**A**) or GIST-T1-NHEJ (**B**) reporter cells were pre-cultured for 48 h with BGJ398 (1 µM), followed by transfection of I-SceI plasmid to induce DNA DSBs, or an empty vector (negative control), for another 96 h. The transfection of pDS-Red2-N1 was used to assess transfection efficiency. Percentages of GFP positive cells arising from HR or NHEJ were determined by flow cytometry. The efficiency of HR and NHEJ was calculated as a ratio of GFP+/DsRed+ cells (the numbers of positive cells are shown in the right quadrants). The representative experiments are shown in A and B. (**C**) Graph illustrating a relative percentage of GFP-positive cells (in %) and SD from six independent experiments.

We found that BGJ398-induced inhibition of FGFR signaling led to the significant decrease of GFP+/DsRed+ ratio in GIST cells stably expressing HR-reporter construct (*p* < 0.01) (Figure 2A). In contrast, BGJ398 treatment did not have an inhibitory impact on this ratio in GIST cells expressing NHEJ-reporter construct (*p* > 0.05) (Figure 2B), thus suggesting that FGFR inhibition in GISTs attenuates homology-mediated DNA repair mechanisms. The average percentages of GFP-positive cells from six independent experiments are depicted in Figure 2C.

### 2.3. Inhibition of FGFR-Signaling Has No Impact on the Processing of Double-Strand Breaks (DSBs)

Given that DNA end resection is known as an early step in HR during which the broken DNA ends are converted into a long stretch of 3′-ended single-stranded DNA (ssDNA) and taking into account that after end resection, the ssDNA is coated with RPA, we initially assessed the impact of FGFR inhibition on formation of ssDNA by BrdU incorporation and RPA foci formation in GIST treated with Dox.

**Figure 3:**
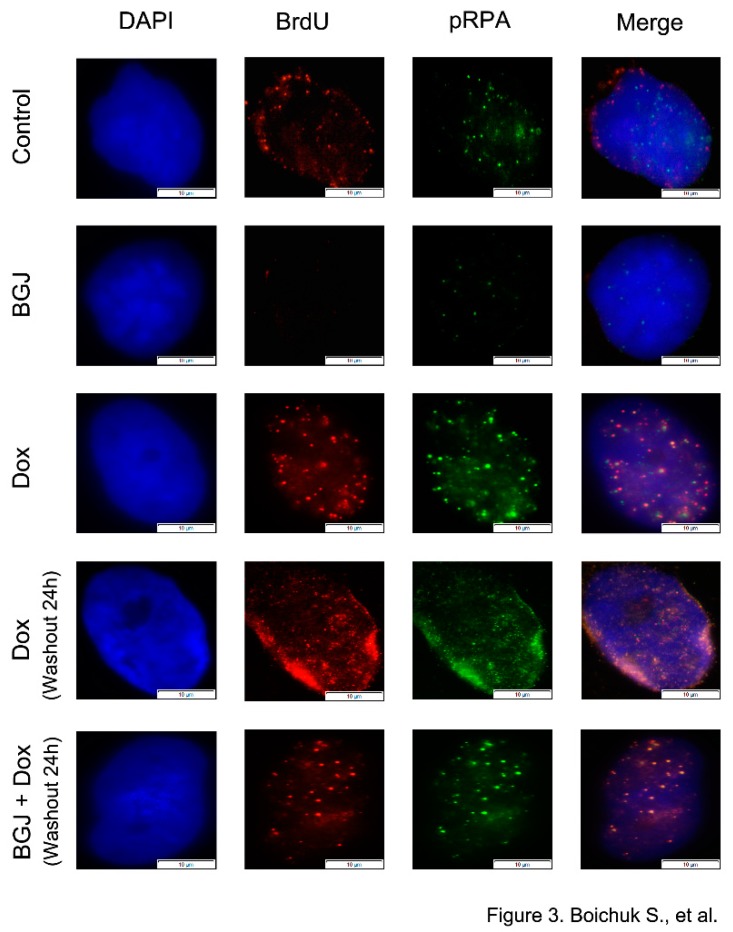
Inhibition of FGF-signaling has no impact on the processing of double-strand breaks (DSBs) and RPA loading. GIST T-1R cells were labeled with 10 µg/mL BrdU for 4 h prior to DNA damage (0.25 µg/mL doxorubicin for 4 h) following washout for 24 h in absence and presence of BGJ398 (1 µM), a selective FGFR1-4 inhibitor. Immunofluorescent detection of incorporated BrdU without denaturation and phosphor-RPA was performed. DAPI staining was used to outline the nucleus. (Scale bars = 10 μm).

As expected, BrdU foci substantially accumulated in GIST after Dox exposure when compared with control (i.e., non-treated) or BGJ398-treated cells (Figure 3). Similarly, a significant proportion of Dox-treated GIST accumulated distinctive phospho-RPA foci. As expected, a large proportion of BrdU foci were co-localized with phospho-RPA after Dox treatment. Importantly, we found that FGFR inhibition has no impact on the numbers of BrdU and pRPA foci in GIST cells treated with Dox (Figure 3–bottom panel), thereby indicating that the initial stages of DDR signaling proceeded normally after FGFR inhibition and resection or another DNA processing event that generates ssDNA remains unaffected during inhibition of FGFR signaling in GIST.

### 2.4. Inhibition of FGFR-Signaling in GIST Impairs RAD51 Loading to DSBs

To perform an effective homology-mediated DNA repair, the DNA DSB ends are needed to be resected to form ssDNAs, which are further coated with replication protein A (RPA). RPA actively recruits and facilitates binding of Rad51 recombinase to the sites of DNA damage and thereby initiates an effective homology-mediated DNA repair [22,23]. Thus, to further explore the regulatory role of FGF-signaling in the homology-mediated DNA repair in GISTs, we performed immunofluorescence staining to determine the effect of FGFR inhibition/depletion on the recruitment of Rad51 to the ssDNAs.

**Figure 4:**
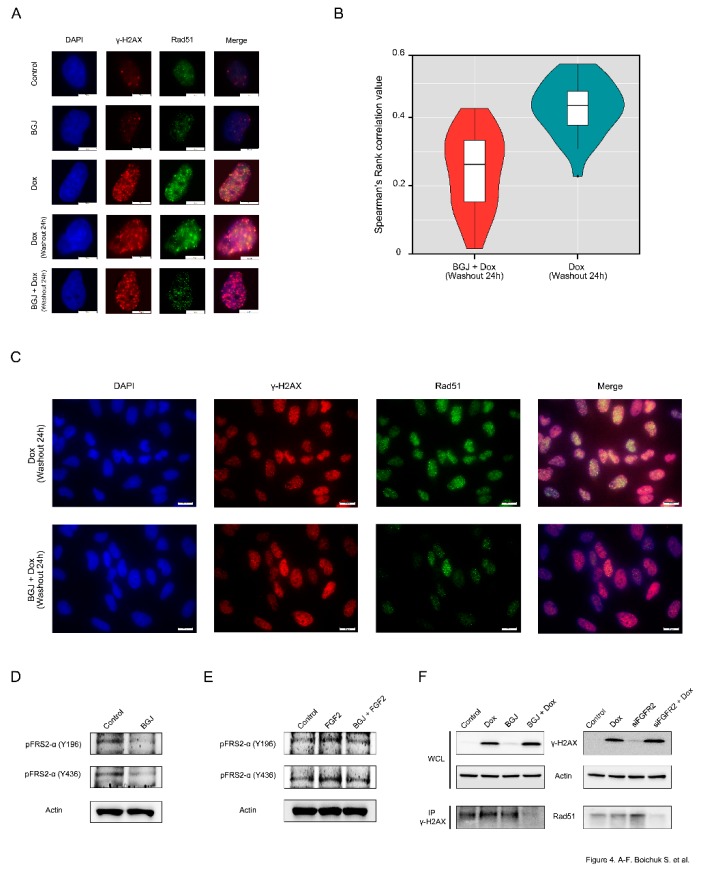
Inhibition of FGF-signaling impairs RAD51 loading to DNA DSBs: (**A**) GIST T-1R cells were pretreated with Dimethyl Sulfoxide (DMSO) (control) or BGJ398 (1 µM) for 48 h prior doxorubicin treatment (0.25 µg/mL) and 4 h later processed for immunofluorescence using Rad51 and γ-H2AX. DAPI staining was used to outline the nucleus (Scale bars = 10 μm). (**B**) Graph illustrating the distributions of Spearman’s rank correlation values between GIST T1-R cells treated with Dox alone (mean = 0.429) or in presence of BGJ398 (mean = 0.245). *p* value = 9.816e-10 (*t*-test). (**C**) Immunofluorescence staining for H2AX and Rad51 was performed on Dox-treated GIST cells after DMSO (control) or BGJ398 treatment. A wider field of cells is shown (scale bars = 20 μm). BGJ398 inhibits FGFR-signaling in IM-resistant and naïve GIST cells: (**D**) BGJ398 abolishes the FGFR-signaling in IM-resistant GIST T-1R cells. The cells were treated with DMSO (control) and BGJ398 (1 μM) for 72 h and were further subjected to immunoblotting with phosphorylated forms of FRS-2 antibodies. (**E**) BGJ398 abrogates FGF-2-induced activation of FGFR-signaling in IM-resistant GIST T-1 cells. The cells were treated with DMSO (control), FGF-2 (100 ng/mL) alone or in presence of BGJ398 (1 μM) for 72 h and were further subjected to immunoblotting with phosphorylated forms of FRS-2 antibodies. (**F**) GIST T-1R cell lysates were immunoprecipitated with γ-H2AX Abs and immunoblotted with Rad51 Abs to demonstrate endogenous complex formation. A whole cell lysate (WCL) was included. Actin was used as a loading control. Inhibition of FGF-signaling was achieved by using BGJ398, a selective FGFR 1-4 inhibitor (left), or siRNA against FGFR2 (right). GIST T-1R cells were pre-treated with BGJ398 1 µM (left) or transfected with siFGFR2 (right) for 48 h before Dox treatment (1 µg/mL for 24 h).

First, we examined whether the Rad51 foci observed in Dox-treated GIST cells are dependent on effective FGFR-signaling. As shown in Figure 4A, Rad51 foci are co-localized with γ-H2AX in GIST cells after Dox treatment. In striking contrast, FGFR inhibition substantially reduced the numbers of Rad51 dots and attenuated the co-localization between Rad51 and γ-H2AX foci after Dox treatment (Figure 4A–*bottom panel*), thus suggesting a failure in the recruitment of Rad51 to DSBs. Spearman’s rank correlation value revealed the decreased co-localization between Rad51 and γ-H2AX foci in FGFR-inhibited GISTs treated with Dox when compared to GIST cells treated with Dox alone (Figure 4B). A larger field of cells stained by immunofluorescence for γ-H2AX/Rad51 confirmed that most of the BGJ398-treated cells exhibited rare, very faint Rad51 dots that were partially mis-localized with γ-H2AX foci (Figure 4C). Similarly, siRNA-mediated depletion of FGFR2 also affected the appearance of Rad51 foci and their co-localization at the DSBs after Dox treatment (data not shown).

The blockage of FGF-signaling in BGJ398-treated GIST T-1R cells was confirmed by immunoblotting, which revealed the lack of phosphorylation of FGFR substrate 2 (FRS2) known as an adaptor protein that plays a critical role in FGF-signaling (Figure 4D). Similarly, FGFR inhibitor indicated above substantially attenuated the phosphorylation of both forms of FRS-2 in IM-naive GIST T-1 cells treated by FGF-2 (Figure 4E), thus confirming a high potency of BGJ398 to inhibit FGF-signaling pathway activated via the autocrine (e.g., by FGF-2 secreted from IM-resistant GISTs) or paracrine (e.g., activated by exogenous FGF-2) mechanisms.

Altogether, this data illustrates that inhibition of FGFR signaling in GISTs attenuates the recruitment of Rad51 to the sites of Dox-induced DNA damage. This was also confirmed by co-immunoprecipitation experiments. For this purpose, the H2AX-interacting complex was first pulled down through immunoprecipitation using γ-H2AX antibodies (Abs). Using the Abs against Rad51, immunoblotting experiments were performed on equal amounts of the immunoprecipitates derived from GIST cells treated with Dox in the presence or absence of BGJ398.

As shown in Figure 4F, the abundance of Rad51 was found in the H2AX-interacting complex in Dox-treated GIST cells but substantially attenuated in GIST cells treated with BGJ398 for 48 h prior Dox exposure (Figure 4F—left bottom panel). Similarly, the knockdown of FGFR2 in Dox-treated GIST substantially decreased the amount of Rad51 precipitated by γ-H2AX-Abs (Figure 4F—right bottom panel), thereby confirming a strong impact of FGFR2-signaling in the recruitment of Rad51 to DSBs in DNA damaging conditions.

### 2.5. FGFR2 Knockdown Sensitizes GISTs to Topoisomerase II Inhibitors and Induces Apoptosis of Cancer Cells

Given that FGFR2 was overexpressed in IM-resistant GIST T-1R cells [16], we sought to examine whether the knockdown of FGFR2 will sensitize tumor cells to DNA-topoisomerase II inhibitors due to attenuation of HR-mediated DNA repair.

**Figure 5:**
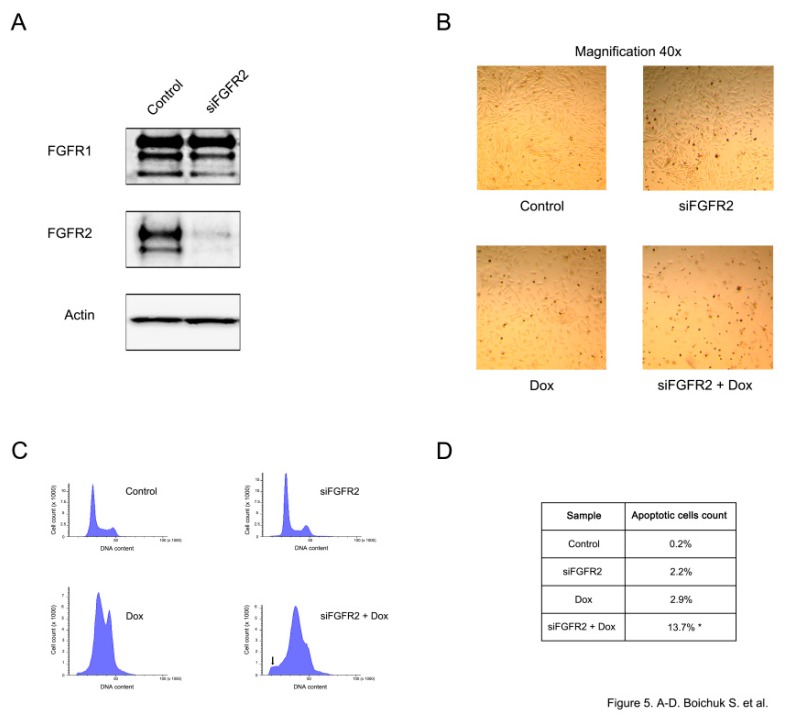
FGFR2 knockdown sensitizes GISTs to topoisomerase II inhibitors and induces their apoptosis. (**A**) GIST T-1R cells were transfected with scrambled siRNA (control) or siRNA targeting FGFR2 for 48 h. The expression of FGFR-1,2 was analyzed by western blotting. Actin was used as a loading control. (**B**) The light microscopy images of doxorubicin (Dox)-treated GIST cells (0.5 µg/mL for 48 h) previously transfected with scrambled siRNA (control) or siFGFR2 for 48 h. (**C**) Representative flow cytometry histograms showing the cell-cycle distribution of GIST T-1R cells transfected with control siRNA or siRNA FGFR2 for 48 h and treated with DMSO or Dox (0.5 µg/mL) for another 48 h. Experiments were conducted in quadruplicate and at least 150,000 cells were counted per experiment. The increase of apoptotic cells was evidenced by a sub-G1 peak in cells depleted of FGFR2 and treated with Dox (noted by arrow). (**D**) Quantification of apoptotic cells was measured by flow cytometry, * *p* < 0.05.

FGFR2 knockdown was confirmed by immunoblotting and demonstrated a substantial reduction of FGFR2 expression, whereas the levels of FGFR1 remained unaffected (Figure 5A). A substantial increase of apoptotic cell death was observed in siFGFR2-transfected GISTs further treated with doxorubicin. This was evidenced by the substantial decrease of cell numbers in GIST culture (Figure 5B) and increased numbers of hypodiploid cells (*p* < 0.05) (Figure 5C,D). 

### 2.6. BGJ398 Sensitizes IM-Resistant GIST to Topoisomerase II Inhibitors via Inhibiting of AKT-Dependent Signaling Pathway

To gain more insights into the molecular mechanisms of BGJ398-induced sensitization of IM-resistant GISTs to Dox, we utilized the AKT- and ERK1/2-inhibitors (MK2206 and U0126, respectively). We found that inhibition of AKT-, but not MAPK-signaling pathway substantially affected GISTs survival after Dox treatment. Indeed, we found ~4-fold difference in the IC50 values for GISTs treated with Dox in the absence or presence of MK2206 (0.94 ± 0.11 and 0.24 ± 0.04 μM, respectively) (*p* < 0.05). A similar effect was observed for another IM-resistant GIST cell line (e.g., GIST430) ~3.5-fold difference in the IC50 values was observed between GIST430s treated with Dox alone and in the presence of MK2206 (2.12 ± 0.36 and 0.58 ± 0.06 μM, respectively) (*p* < 0.05). In contrast, U0126 has a minor effect on sensitizing of both IM-resistant GIST cell lines to Dox, thereby indicating that inhibition of FGF-signaling attenuates the viability of Dox-treated GISTs predominantly via AKT-dependent pathway.

Altogether, this data illustrates that inhibition of AKT-signaling is a predominant molecular mechanism involved in the sensitization of FGFR-inhibited GISTs to topoisomerase II inhibitors.

### 2.7. Exogenous FGF-2 Reduces GIST Sensitivity to Topoisomerase II Inhibitors

To test the ability of FGF2 to reduce GIST sensitivity to topoisomerase II inhibitors (e.g., doxorubicin and etoposide), MTS-based assay was performed for GIST cells cultured with 8 different concentrations of topoisomerase II inhibitors indicated above in the absence (control) or presence of FGF-2 (20 ng/mL). For these experimental settings, we utilized the serum-starved cells to exclude an impact of the growth factors, including FGF-2, present in the fetal bovine serum. We observed that FGF-2 treatment of serum-starved GIST cells increased IC50 values for Dox and Eto (1.5 and 2.4-fold, respectively), thereby revealing the importance of FGF-signaling in the survival of GIST cells treated with topoisomerase II inhibitors. For example, the IC50 values for Dox alone and in the presence of FGF-2 (0.48 ± 0.04 and 0.71 ± 0.06 μM, respectively) (*p* < 0.05). Similarly, IC50 for Eto in absence and presence of FGF-2 were 20.32 ± 3.52 and 47.51 ± 5.01 μM, respectively) (*p* < 0.05). Of note, FGF-2 alone has a minor stimulatory effect on GIST cell growth, thereby excluding the possibility of the primary growth effect mediated by FGF2 in these experimental settings.

## 3. Discussion

Fibroblast growth factors (FGFs) and their receptors (FGFR1-4) regulate a broad spectrum of physiologic cellular processes, such as differentiation, proliferation, survival, migration, and angiogenesis. Meanwhile, an aberrant FGFR-signaling is well-documented for a wide spectrum of human malignancies and plays an important role in carcinogenesis, tumor development, and progression, thereby validating this pathway as an attractive target for cancer therapy.

For example, FGFR-activating mutations are frequently observed in urothelial bladder carcinoma, endometrial cancer, rhabdomyosarcoma etc., and FGFR-mutant cancer cells were found sensitive to selective FGFR inhibitors in vitro and in vivo [24,25,26,27,28,29]. FGFR1-4 amplifications were detected in squamous non–small cell lung carcinoma [30,31], osteosarcoma, small cell lung carcinoma, gastric and breast cancer, and were associated with tumor sensitivity to FGFR inhibitors in the preclinical in vivo models. In addition to activating FGFR mutations and/or FGFR amplifications, aberrant FGFR-signaling in cancer cells might be a result of the increased activities of autocrine/paracrine loops leading to increased secretion of FGFR ligands (i.e., FGFs) by cancer or stromal cells, thus promoting cancer cell survival and proliferation. Increased activity of autocrine/paracrine loops in tumor cells might also be important in tumor pathogenesis due to the well-known abilities of FGFs, and especially FGF2, to stimulate angiogenesis [32,33] and thus promote tumor growth, invasion and disease progression. Finally, in addition to direct pro-angiogenic effects of FGFR-signaling, activation of this pathway might also mediate the activity of VEGFR-signaling and thus synergizes with VEGFR and platelet-derived growth factor receptor (PDGFR)-mediated pathways to promote tumor neo-angiogenesis [34,35]. An increased activity of autocrine/paracrine FGF2/FGFR-mediated loops were proofed for the multiple types of malignancies, including endometrial, bladder, non-small cell lung cancer, breast, multiple myeloma, prostate, gastric and colon cancers.

Importantly, an increased FGFR expression and/or activity has also been reported to play a role in tumor resistance to conventional and targeted-based anti-cancer therapies. For example, increased FGFR2 and FGFR4 expression were associated with poor response to neoadjuvant chemoradiation in colorectal cancer patients [36,37]. Targeting of FGFR1 was found to increase radiation-induced killing of mesothelioma cells [38], whereas inhibition of FGFR3 enhanced radiosensitivity of squamous cell carcinomas [39]. FGFR1 amplification was found to promote the resistance of luminal B type breast cancer to hydroxytamoxifen, whereas inhibition of FGF-signaling by siFGFR1 abrogated this resistance [40]. Currently ongoing clinical trial (NCT01202591) is evaluating the effectiveness of the combination of FGFR-inhibitor and endocrine therapy for the patients with estrogen-positive (ER+) breast cancer. Similarly, FGFR3 activation was proposed as a mechanism of cancer cell resistance to targeted-based therapy. This was evidenced for KRAS wild-type squamous cell carcinomas that acquired resistance to cetuximab and proofed for BRAF (V600E)-mutant melanoma cells that became resistant to vemurafenib [41,42].

Taken together, this data illustrates that aberrant FGFR-signaling plays an important role in carcinogenesis, tumor development, progression and mediate an acquired resistance of cancer cells to the current therapeutic regimens thus affecting the effectiveness of cancer therapy and prognosis.

However, to date, little is known about the role of FGFR-signaling pathway in GISTs biology and sensitivity to targeted-based therapy and chemotherapy. For example, Li F., with co-authors illustrated an increased expression of FGF2 and FGFR1 in primary GIST samples, thus suggesting the increased activities of autocrine/paracrine loops [36]. Importantly, the combined inhibition of KIT- and FGFR-signaling increased growth inhibition in IM-sensitive GIST cells in vitro and in vivo in patient- derived GIST xenografts [43]. Another report indicated that FGF2 expression was increased in IM- resistant GIST cells and KIT- and FGFR3 inhibitors synergized to inhibit GISTs growth in vitro [44]. Our recent data illustrated an increased expression of phospho-FGFR2 in IM-resistant GIST, whereas expression of pKIT was decreased, thus illustrating the RTK switch (loss of pKIT/gain of pFGFR2) over development of IM resistance [16]. Indeed, we further observed that inhibition of FGF-signaling by using the selective FGFR1-3 inhibitor or depletion of FGFR2 by corresponding siRNAs substantially enhanced the cytotoxic and pro-apoptotic effects of IM in IM-resistant GIST cells (unpublished data). Moreover, BGJ398, a selective FGFR1-3 inhibitor, has a high potency to sensitize IM-resistant GIST cells to the topoisomerase II inhibitors, doxorubicin and etoposide. A substantial decrease of cell viability, proliferation and growth was found in IM-resistant GIST cells simultaneously treated with FGFR- and DNA-topoisomerase II inhibitors [18]. Based on this data, we wondered whether inhibition of FGFR- signaling might have an impact on DNA damage signaling and repair in GIST cells, especially on tumor cells that acquired IM- resistant phenotype over IM-based therapy. To our opinion, the last one is important due to the limited therapeutic options for GIST patients who developed the disease progression despite IM-based targeted therapy and having an advanced, metastatic and/or recurrent form of the disease.

Given that DNA-topoisomerase II inhibitors induce DNA damage, including base damage, single-strand breaks (SSB), double-strand breaks (DSB) and unrepaired or mis-repaired DSBs are lethal and taking into account that γ-H2AX is a common marker of DSBs, we initially examined whether doxorubicin-induced DSBs in GIST remained unrepaired after the inhibition of FGF-signaling. Indeed, γ-H2AX expression in GIST retained for the much longer period of time when the cells were cultured in presence of BGJ398 after Dox was washed out from the cell culture (Figure 1C,D). An increased tail moment in GIST cells cultured with FGFR inhibitor after Dox treatment (Figure 1A,B) revealed the unrepaired dox-induced DSBs and suggested that inhibition of FGF-signaling might have a direct impact on DSB repair.

Given that mammalian cells utilize two major pathways to repair DSBs (e.g., homologous recombination (HR) and non-homologous end-joining (NHEJ), we hypothesized that an ability of BGJ398, a selective FGFR inhibitor, to sensitize GISTs to doxorubicin and/or etoposide might be due to inhibition of DSBs repair pathway(s) indicated above. By utilizing GFP-based reporter constructs, having GFP gene containing recognition sites for a I-SceI endonuclease for induction of DSBs, we showed that inhibition of FGFR-signaling in GISTs substantially attenuates a homology-mediated DNA repair, whereas NHEJ-mediated repair pathway remained unaffected in these experimental settings (Figure 2).

Since DNA end resection is known as an early step in homology-mediated repair leading to the conversion of broken DNA ends into a long stretch of 3′-ended single-stranded DNA (ssDNA), we initially examined whether BGJ398-induced inhibition of HR is due to either absence of ssDNA or a defect in the loading of RPA to ssDNA. Data shown in Figure 3 illustrates that inhibition of FGF-signaling did not have a negative impact on these processes, which was evidenced by similar numbers of BrdU- and pRPA-positive foci in Dox-treated cells cultures in absence and presence of FGFR inhibitor. Moreover, in Dox-treated GIST cultured in presence of FGFR inhibitor the BrdU- and pRPA-positive foci were highly co-localized, thereby illustrating a perfect loading of RPA to ssDNA. Next, we examined whether inhibition of FGF-signaling has a negative impact on the recruitment of Rad51 recombinase into the DSBs. Strikingly, our immunofluorescence data shown on Figure 4A illustrates that inhibition of FGF-signaling in GIST decreases the loading of Rad51 to the sites of DNA DSBs that were marked as γ-H2AX-positive foci. This was also confirmed by a decreased expression of Rad51 recombinase immunoprecipitated by γ-H2AX Abs (Figure 4B).

The molecular mechanisms responsible for BGJ398-induced inhibition of Rad51 loading to the DSBs in GIST remain to be further elucidated. One possibility is due to the potential dysregulation of chromatin-bound PTEN/Ki-67 complexes that were recently shown to be critical in the recruitment of Rad51 into the sites of DNA damage to promote DNA repair. *Ma J*. with co-authors showed that phosphorylation of PTEN on Tyr^240^ is rapidly elevated in response to ionizing radiation (IR) and bound to chromatin via interaction with Ki-67 in glioblastoma (GBM) cells. Strikingly, this was found critical for the recruitment of Rad51 to the DSBs to promote DNA repair. Important, inhibition of FGFR phosphorylation abrogated PTEN phosphorylation and its consequent chromatin interaction with Ki-67 in response to IR-induced DNA damage, thereby enhancing GBM radiosensitivity through attenuated DNA repair [45]. This is also in consistency with our data illustrating that *FGFR2* knockout effectively sensitized GIST to the low doses of DNA-topoisomerase II inhibitors and promoted apoptotic cells death (Figure 5).

Taken together, our data provides the evidence that inhibition of FGFR-signaling in IM-resistant GISTs sensitizes them to DNA-damaging agents via attenuating of the homology-mediated DNA DSBs repair. Therefore, this approach may increase the efficiency of DNA damage-associated therapies for cancer patients and might be helpful to overcome tumor chemoresistance. This is also in consistency with the reports indicating a significant impact of FGF-signaling inhibition on tumor cells proliferation and sensitivity to the chemotherapeutic agents in vitro and in vivo. For example, *Cole C.* with the co-authors found the inhibition of FGFR2 and/or FGF-3 and -7 substantially reduced the proliferation of ovarian cancer cells and reduced the IC50 for cisplatin in vitro. In vivo studies revealed that FGFR2 inhibition slowed tumor growth and augmented the cytotoxic effects of cisplatin [46]. FGFR inhibitor PD173074 potentiated the effects of cisplatin in small cell lung cancer cells, thus illustrating that inhibition of FGF-signaling can augment the effects of chemotherapeutic agents [47]. A potential therapeutic benefit of combining an FGFR inhibitor PD173074 with the standard chemotherapeutic agents (e.g., paclitaxel or doxorubicin) was also observed for patients with endometrial cancer, especially with FGFR2-mutation-positive tumors [48].

## 4. Materials and Methods

### 4.1. Chemical Compounds

Imatinib mesylate (IM), MK2206, U0126 and BGJ398 were obtained from SelleckChem (Houston, TX, USA), Doxorubicin (Dox) and G418 were purchased from Sigma-Aldrich (St. Louis, MO, USA).

### 4.2. Antibodies

Primary antibodies used for immunoblotting, co-immunoprecipitation and immunofluorescence were as follows: γ-H2AX S139, Rad51 (Santa Cruz Biotechnology, Santa Cruz, CA, USA; Abcam, Burlingame, CA, USA), pRPA32 S4/S8 (Bethyl Laboratories, Montgomery, TX, USA), anti-BrdU (Becton Dickinson Biosciences, Franklin Lakes, NJ, USA), phospho-FRS2α Y196 and Y436, FGFR1, 2 (Cell Signaling, Danvers, MA, USA), beta-actin (GenScript, Piscataway, NJ, USA).

### 4.3. Cell Lines and Culture Conditions

GIST T-1 cell line was a generous gift of Dr. Taguchi (Kochi University, Kochi, Japan). It was established from untreated metastatic GIST and contained heterozygous 57-base pair deletion (V570-Y578) in KIT exon 11 [49]. IM-resistant GIST T-1R subline established in our laboratory after a continuous induction from 0.4 nM to 1000 nM IM in a stepwise increasing concentration manner [16]. GIST T-1 and T-1R cells were maintained in RPMI-1640 medium supplemented with 15% fetal bovine serum (Thermo Fisher Scientific, Waltham, MA, USA); 1% L-glutamine, 50 U/mL penicillin, 50 µg/mL streptomycin (PanEco company, Moscow, Russia). The cell lines indicated above were cultured in a humidified atmosphere of 5% CO2 at 37 °C (Lam Systems, Miass, Russia).

### 4.4. Western Blotting and Co-Immunoprecipitation (Co-IP)

For Western blotting analysis, whole-cell extracts were prepared by scrapping the cells growing as monolayer into RIPA buffer (25 mM Tris-HCl pH 7.6, 150 mM NaCl, 5 mM EDTA, 1% NP-40, 1% sodium deoxycholate, 0.1% SDS), supplemented with protease and phosphatase inhibitors. The cellular lysates were incubated for 1 h at 4 °C and then clarified by centrifugation for 30 min at 13,000 rpm at 4 °C. Protein concentrations were measured by the Bradford assay. The samples containing 30 μg of protein were resolved on 4 to 12% Bis-Tris or 3 to 8% Tris- acetate NuPAGE gels (Invitrogen, Carlsbad, CA, USA), transferred to a nitrocellulose membrane (Bio-Rad, Hercules, CA, USA), probed with specific antibody, and visualized by enhanced chemiluminescence (Western Lightning Plus-ECL reagent, Perkin Elmer, Waltham, MA, USA). Densitometric analysis of western blotting images was performed by using the NIH Image J software (Bethesda, MD, USA). For Co-IP, cells after treatment were lysed by TEB buffer (50 mM Tris-HCl pH 7.5, 150 mM NaCl, 1% NP-40, 10% glycerol), supplemented with protease and phosphatase inhibitors. The lysates were cleared by centrifugation and further cultured with the corresponding Abs overnight (rotating devise at 4 °C). The samples were further incubated with protein A and G Sepharose beads (Santa Cruz Biotechnology, Santa Cruz, CA, USA) for 1 h (rotating devise at 4 °C). The beads were washed 3 times with TEB buffer and resolved by SDS-PAGE. Then 1X sample buffer was added to the resolved precipitates and the mixture was boiled for 5 min. Subsequently, Western blotting was performed as indicated above.

### 4.5. Immunofluorescence Staining

Cells were seeded on glass coverslips coated with poly-L-lysine (Sigma-Aldrich, St. Louis, MO, USA) and allowed to attach for 48 h before treatment. After washing with ice-cold PBS, cells were fixed in 4% paraformaldehyde in PBS for 30 min at 4 °C and further permeabilized with 0.5% Triton X-100 for 5 min. In case of BrdU and pRPA staining, before the cells were fixed, the slides were pre-extracted with CSK buffer [10 mM piperazine-*N*,*N*′-bis (2-ethanesulfonic acid) (PIPES; pH 6.8), 100 mM NaCl, 300 mM sucrose, 3 mM MgCl_2_, 1 mM EGTA, 0.5% Triton X-100] for 5 min on ice, followed by incubation in cytoskeleton stripping buffer (10 mM Tris-HCl [pH 7.4], 10 mM NaCl, 3 mM MgCl_2_, 1% Tween 40, 0.5% sodium deoxycholate) for 5 min on ice [50]. After 30 min of blocking with 10% normal goat serum in PBS, cells were washed and incubated with primary antibodies for overnight at 4 °C. Next day the cells were washed with PBS, incubated with Alexa Fluor 488-, or Texas Red-conjugated secondary antibodies (Invitrogen, Carlsbad, CA, USA) for 30 min at room temperature in the dark. After brief DAPI (Sigma-Aldrich, St. Louis, MO, USA) staining, the cover slips were mounted on glass slides and cells visualized on an Olympus BX63 fluorescence microscope. Images were captured using a Spot advanced imaging system.

### 4.6. Image Quantification and Registration

Cell were seeded for the 6-well plates and allowed to grow for 48 h before treatment. The cells were fixed, permeabilized and stained as shown above. Plates were imaged with a 10 × objective using a Cytell Cell Imaging System (GE Healthcare, Buckinghamshire, UK). MyBioApp Protocol with specified parameters was created to acquire the data and to quantify the signal intensity in nuclei, cytoplasm, and in whole cells. DAPI (blue channel) was used for the nuclear masks, whereas Alexa Fluor 647 (red channel) was used for γ-H2AX-staining. Graphics illustrating the intensity of the nuclear γ-H2AX-staining were automatically generated by using MyBioApp Protocol and then passed to MS Exel for further processing and analysis.

### 4.7. FGFR2 Silencing Using siRNA

For FGFR2 knockdown experiments, On-Target Plus Smartpool siRNA targeting FGFR2 (Dharmacon RNA Technologies, Lafayette, CO, USA) was diluted in Opti-MEM (Thermo Fisher Scientific, Waltham, MA, USA) and complexed with RNAimax (Invitrogen, Carlsbad, CA, USA) to a final concentration of 40 nM oligonucleotides. The oligomer-RNAimax complex was added to the culture medium. Cells transfected with Non-Target plus si control pool were used as a negative control. After 48–72 h, cell lysates were subjected to SDS-PAGE and immunoblotting for validation of successful knockdown.

### 4.8. Analysis of HR and NHEJ Efficiency by Flow Cytometry

To examine the impact of FGFR inhibition on the efficiency of HR- and NHEJ-mediated pathways in GIST, we previously generated GIST T1-R sublines stably expressing HR- or NHEJ-based reporter constructs (kindly provided by Vera Gorbunova, University of Rochester, Rochester, NY USA). The efficiency of HR and NHEJ in GIST cells were analyzed by FACS according the standard protocol shown before [16]. Briefly, GIST cells containing the chromosomally integrated HR- or NHEJ-based reporter constructs were transfected with I-SceI-expressing plasmid (to induce DSBs) and pDsRed2-N1 (Clontech Laboratories, Mountain View, CA, USA) as a transfection efficiency control. The cells were transfected by using Amaxa Nucleofector following manufacturer’s recommendations and using the program T-23. Four days after transfection the control and I-SceI transfected cells were harvested and analyzed by Flow Cytometer BD FACSCanto II using BD FACSDiva Software (Becton Dickinson Biosciences, Franklin Lakes, NJ, USA). The percentage of GFP+ cells was to assess the efficiency of DNA DSB repair, whereas the percentage of DsRed+ cells was used for transfection efficiency. The ratio between GFP+ cells and DsRed+ cells (the percentages of the double-positive cells) was used to calculate the relative efficiency of DNA DSB repair in GIST cells.

### 4.9. Analysis of DNA Strand Break Induction (Comet Assay)

Formation of DNA strand breaks was assayed by the alkaline comet assay. Comets were visualized by microscopy and quantified by determination of the percentage of DNA in the tail. The alkaline comet assay parameters were evaluated by the CaspLab software version 1.2.3. 50 nuclei were evaluated per treatment.

### 4.10. Cell Cycle Analysis and Light Microscopy

Cell cycle distribution was studied by measuring the amount of propidium iodide (PI)-labeled DNA in ethanol-fixed cells. In brief, cells were harvested by trypsinization, washed twice with PBS, containing 1% fetal bovine serum (FBS), and fixed with ice-cold 70% ethanol. After fixation, cells were washed twice with PBS (1% FBS) and then re-suspended in 1 mL PI staining solution (50 μg/mL PI, 10 mM Tris-HCl [pH = 7.4], 5 mM MgCl_2_, 10 µg/mL RNase) and incubated at 37 °C in the dark for 30 min. PI and RNase were purchased from Sigma-Aldrich (St. Louis, MO, USA). Cell cycle analysis was conducted on a Flow Cytometer BD FACS Canto II using BD FACS Diva Software (Becton Dickinson Biosciences, Franklin Lakes, NJ, USA). Cell light microscopy was carried out using a digital microscope Leica DFC420 (Leica Microsystems, Mannheim, Germany).

### 4.11. Co-Localization Analysis

100 × images were processed by Fiji Software (Laboratory for Optical and Computational Instrumentation (LOCI), University of Wisconsin, Madison, WI, USA). The images were subjected to deconvolution by using Parallel Spectral Deconvolution plugin, regions of interest (ROI), representing the nuclei, were selected, and co-localization analysis was performed using Coloc2 plugin. To choose the appropriate method of co-localization analysis, the distributions of pixel intensity values of each ROI were assessed for normality by using Kolmogorov-Smirnov test in R software, version 3.6.0 (R Foundation for Statistical Computing, Vienna, Austria. URL https://www.R-project.org/), and Spearman’s rank correlation value was selected for further analysis. Total amount of 30 nuclei were processed for each experimental setting, and the means of Spearman’s values for each group were compared by using t-test in R software.

### 4.12. Statistics

All the experiments were repeated a minimum of 3 times. The results are presented as the mean ± standard error (SE) for each group. Differences were considered significant at *p* < 0.05.
